# Efficient peroxymonosulfate activation by magnesium-doped Co_3_O_4_ for thiacloprid degradation: regulation of Co^2+^/Co^3+^ ratios and degradation mechanism

**DOI:** 10.1039/d5ra04080a

**Published:** 2025-08-28

**Authors:** Hui Fui, Xinran Ma, Yiping Huang, Shiyao Xi, Zhandong Ren, Yuchan Zhu

**Affiliations:** a Hubei Province Key Laboratory of Agricultural Waste Resource Utilization, Wuhan Polytechnic University Wuhan 430023 China feihui509@163.com; b School of Chemistry and Environmental Engineering, Wuhan Polytechnic University Wuhan 430023 China

## Abstract

AS a low-cost and high-performance catalyst, spinel cobalt oxide (Co_3_O_4_) has two different catalytic active sites (tetrahedral Co^2+^ and octahedral Co^3+^) to drive the activation of peroxymonosulfate (PMS) through Co^2+^/Co^3+^ redox cycle. Tuning Co^2+^/Co^3+^ atomic ratio on the surface of Co_3_O_4_ for the construction of a synergy in the Co^2+^/Co^3+^ redox cycle might be an effective way to further boost PMS activation performance of Co_3_O_4_ catalyst. Herein, we suggested a metal-doping strategy to regulate Co^2+^/Co^3+^ atomic ratio of Co_3_O_4_ by partially substituting Co^2+^ with inert Mg^2+^ and formed a series of Mg doped Co_3_O_4_ (MCO) catalysts. Structural characterizations and experimental investigations demonstrated that Mg doping did not change Co_3_O_4_ host lattice and particle morphology, but could manipulate surface Co^2+^/Co^3+^ atomic ratio of Co_3_O_4_ for an improved PMS activation. The optimal MCO catalysts (MCO-0.2) with the suitable Co^2+^/Co^3+^ atomic ratios (1.13) exhibited the excellent thiacloprid (THIA) degradation performance through PMS activation, and the apparent degradation rate constant (0.2835 min^−1^) was highly outperformed that of pure Co_3_O_4_ (0.09555 min^−1^) and other similar cobalt-based catalysts. The optimal THIA degradation conditions might be: catalyst dose 100 mg L^−1^, PMS concentration 0.8 mM, pH 7 and THIA concentration 20 mg L^−1^. Quenching experiments and electron paramagnetic resonance (EPR) characterizations suggested SO_4_˙^−^, HO˙ and ^1^O_2_ were all involved in THIA degradation during the MCO-0.2/PMS process. Furthermore, the steady-state concentrations of these reactive species and their relative contributions to THIA degradation were also calculated by combining a kinetic model and a series of probe compound-based experiments. The results indicated that SO_4_˙^−^ and HO˙ were generated at lower steady-state concentrations than that of ^1^O_2_, but they dominated THIA abatement during the MCO-0.2/PMS process. This study presented new insights into the construction of efficient PMS activator and a mechanistic understanding for PMS-mediated reaction.

## Abstract 2

A magnesium doping strategy on regulating Co^2+^/Co^3+^ atomic ratio over Co_3_O_4_ for the construction of a synergy in the Co^2+^/Co^3+^ redox cycle highly boosted PMS activation performance of Co_3_O_4_ catalyst.

## Graphical abstract

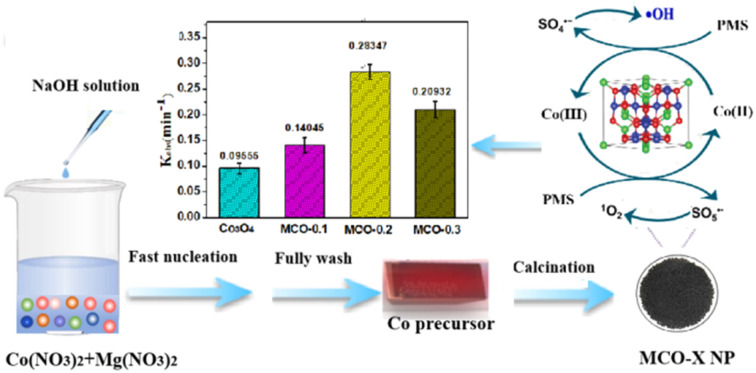

## 1. Introduction

Refractory organic pollutants (*e.g.*, pesticides, antibiotics and industrial chemicals) in wastewater constitute a serious threat to the ecosystem and human health.^1–3^ For eliminating these organic pollutants from wastewater, peroxymonosulfate-based advanced oxidation processes (PMS-AOPs) have been extensively studied and recognized as a promising approach^4–6^ during the past few years. In PMS-AOPs, reactive species (RS) originated from PMS activation is crucial to accelerate these organic pollutants degradation.^7–9^ Therefore, many catalysts, including some transition metals (Co, Fe, Cu and Mn),^10–13^ carbonaceous materials and their composites,^14,15^ have been developed for efficient PMS activation during the past few years. Of all these catalysts, Co-based heterogeneous catalysts (*e.g.*, metal ions, oxides, hydroxides and Co-containing single atomic catalysts)^16–20^ have drawn extensive attention because they are amongst the most active PMS activator and can be recycled to minimize environmental impact.^21,22^

Among various Co-based catalysts, Co_3_O_4_ stands out as a low-cost catalyst and was widely used in different oxidation reaction systems.^23,24^ Normal Co_3_O_4_ catalysts with two different Co sites in a spinel structure, where Co^2+^ is bonded to four neighboring oxygen atoms at tetrahedral sites and Co^3+^ is bonded to six neighboring oxygen atoms at the octahedral sites, have been demonstrated to be efficient for PMS activation.^25,26^ Previous studies^27,28^ have revealed that both tetrahedral Co^2+^ and octahedral Co^3+^ were the efficient active sites of PMS activation in PMS-AOPs over the Co_3_O_4_ catalysts. Specifically, two reactions (the reductive reaction of PMS and the oxidative reaction of PMS) should take place simultaneously to generate radicals continuously through a Co^2+^/Co^3+^ redox cycle (eqn (1) and (2)).^27,28^

The oxidative reaction of PMS:

1 Co^2+^ + HSO_5_^−^ → Co^3+^ + SO_4_˙^−^ + OH^−^

The reductive reaction of PMS:

2 Co^3+^ + HSO_5_^−^ → Co^2+^ + SO_5_˙^−^ + H^+^

Undoubtedly, the different density of Co^2+^/Co^3+^ on the surface of Co_3_O_4_ catalysts might be resulted in the different capacity for the circulation of Co^2+^/Co^3+^ and further vigorously affect the activity of Co_3_O_4_ catalysts.^29^ Moreover, the different density of Co^2+^/Co^3+^ on the surface could also change atomic arrangements and electronic structures of Co_3_O_4_ to favour Co^2+^/Co^3+^ redox cycle.^30,31^ Thus, tuning the atomic ratios of Co^2+^/Co^3+^ exposed on the surface of Co_3_O_4_-based catalytic materials could be a reasonable way to construct a synergistic effect of Co^2+^ and Co^3+^ in the Co^2+^/Co^3+^ redox cycle for an improved PMS activation.

Metal doping was a simple and efficient approach to engineer surface tetrahedral Co^2+^ and octahedral Co^3+^ of Co_3_O_4_ for manipulating the ratio of Co^2+^/Co^3+^ of Co_3_O_4_ by the substitution of tetrahedral Co^2+^ and octahedral Co^3+^ with the corresponding valence states of inactive metal.^32,33^ For example, Dong's group^17^ reported the synthesis of Al doped Co_3_O_4_ catalysts by incorporating inert Al^3+^ ion into the lattice of Co_3_O_4_. They found that Al incorporation could partly replace octahedral Co^3+^ and modify the ratio of Co^2+^/Co^3+^ in Co_3_O_4_ for an improved performance of PMS activation. While previous studies^34^ have demonstrated octahedral Co^3+^ possessed a high standard reduction potential (*E*_0_(Co^3+^/Co^2+^) = 1.92 V) and was notably more active than tetrahedral Co^2+^ for OER. The significant roles for pollutant abatement of octahedral Co^3+^ were also confirmed during the PMS-AOPs.^35^ Therefore, the engineering of tetrahedral Co^2+^ by the doping of divalent metal ion (Zn^2+^ and Mg^2+^) might be an effective way to regulate the atomic ratio of Co^2+^/Co^3+^ of Co_3_O_4_ for an improved PMS activation.^10,20^ While Mg^2+^ has a similar ionic radius to that of Co^2+^ (0.72 Å *vs.* 0.74 Å).^28^ The precise substitution of tetrahedral Co^2+^ with inert Mg^2^ could be easily realized for the regulation of Co^2+^/Co^3+^ ratio in the spinel structure of Co_3_O_4_, but might not change Co_3_O_4_ host lattice,^28,29^ which facilitated us to judge the important role of Co^2+^/Co^3+^ ratio in PMS activation, however it has not been reported yet.

Hence, a magnesium doping strategy was developed to manipulate Co^2+^/Co^3+^ ratio in the cobalt spinel for an enhanced PMS activation, and obtained a series of Mg doped Co_3_O_4_ (MCO) catalysts. Optimal MCO catalysts (MCO-0.2) with suitable Co^2+^/Co^3+^ atomic ratios possessed superior activities for THIA degradation during PMS-AOPs, and the rate constant (0.2835 min^−1^) was 2.97 folds faster than that of pure Co_3_O_4_ (0.0955 min^−1^). To further elucidate the degradation mechanisms, the formation of reactive species in the MCO-0.2/PMS process was verified by scavenger tests and EPR characterizations. Moreover, the steady-state concentrations of these RS were quantified through a kinetic model and several probe experiments. Using the newly measured kinetic data, the relative contributions of these RS to THIA degradation were thus determined. This study might present valuable design guide of cobalt-based catalysts with regulated Co^2+^/Co^3+^ ratio for environmental applications.

## 2. Experimental procedures

### 2.1. Chemicals and materials

Thiacloprid (THIA), sodium hydroxide (NaOH), atrazine (ATZ), cobalt nitrate hexahydrate (Co(NO_3_)_2_·6H_2_O), sodium bicarbonate (NaHCO_3_), methanol (MeOH), magnesium nitrate dihydrate (Mg(NO_3_)_2_·2H_2_O), chloramphenicol (CAP) and *tert*-butyl alcohol (TBA) were obtained from Sinopharm Chemical Reagent Co. Ltd. Potassium peroxymonosulfate (PMS), sodium chloride (NaCl), sulfuric acid (H_2_SO_4_), sodium nitrate (NaNO_3_), disodium hydrogen phosphate (Na_2_HPO_4_), sodium thiosulfate (Na_2_S_2_O_3_), furfuryl alcohol (FFA), methyl phenyl sulfone (PMSO_2_), metronidazole (MTZ), methyl phenyl sulfoxide (PMSO), commercial nano-Co_3_O_4_ and MgCo_2_O_4_ were purchased from Aladdin Company, China. All chemical reagents were used without further purification. Deionized water (DI water) was used throughout the whole experiments.

### 2.2. Catalyst synthesis

The synthesis of MCO catalysts involved the pre-synthesis of Co-based precursors and subsequently calcining them under air atmosphere. The obtained catalysts were labelled MCO-*X*, where *X* indicated the nominal molar ratio of Mg/(Co + Mg) in the catalysts. Taking MCO-0.2 as an example, 1.5 mmol Mg(NO_3_)_2_·2H_2_O and 6 mmol Co(NO_3_)_2_·6H_2_O were mixed in 100 mL DI water. Subsequently, the solution pH was adjusted to 11 by dropwise addition of 0.1 M NaOH solution under continuous stirring. The above solution was centrifugated at 10 000 rpm for 10 min, and the obtained precipitate was resuspended in a small volume of DI water, repeating the procedure until the suspension pH reached 8. After rinsing with ethanol several times, the precipitate was dried at 80 °C for 24 h, and then thermally treated at 350 °C for 3 h in air atmosphere to obtain the final product. Similarly, Co_3_O_4_ (Co(NO_3_)_2_·6H_2_O: 7.5 mmol), MCO-0.1 (Mg(NO_3_)_2_·2H_2_O: 0.75 mmol, Co(NO_3_)_2_·6H_2_O: 6.75 mmol), MCO-0.3 (Mg(NO_3_)_2_·2H_2_O: 2.25 mmol, Co(NO_3_)_2_·6H_2_O: 5.25 mmol) were prepared as above using different doses of Mg(NO_3_)_2_·2H_2_O and Co(NO_3_)_2_·6H_2_O. For comparison, ZnCo_2_O_4_ was prepared through the above procedure with appropriate doses of Zn(NO_3_)_2_·2H_2_O and Co(NO_3_)_2_·6H_2_O. β-Co(OH)_2_ were synthesized with same method as that of Co_3_O_4_ without thermal treatment.

### 2.3. Catalytic performance

The activities of Co_3_O_4_ and MCO catalysts were checked in 100 mL THIA solution with mechanically agitating. Specifically, the catalysts were first spiked into THIA solution and further stirred until adsorption/desorption equilibrium, followed by the initiation of the reaction with the introduction of 0.2 mM PMS. Periodically, 2 mL samples were filtered through a 0.22 μm filter and immediately quenched with Na_2_S_2_O_3_ solution, followed by component analysis. The pH value of the reaction system was under control utilizing 0.1 M NaOH or H_2_SO_4_. The presence of reactive species (RS) in THIA degradation was evaluated by scavenger tests, where TBA, MeOH, DMSO and FFA were used as RS quenchers and spiked into the system before PMS addition. In addition, the depletion experiments of several probe compounds (including CAP, ATZ and MTZ) were performed as the same conditions as that of THIA abatement to measure RS exposures during the MCO-0.2/PMS process. Moreover, the reusability of MCO-0.2 catalysts was investigated through recycle tests. After each run, MCO-0.2 water and then reused under the same experimental conditions (details provided in Text S1).

### 2.4. Analytical methods

The crystalline structures of the synthetized catalysts were detected by a Bruker D8 Advance X-ray diffractometer (XRD) using Cu Ka radiation (*λ* = 1.5418 Å) with 2-theta range of 10–80° and scanning rate of 10° s^−1^. The micro-morphologies of pure Co_3_O_4_ and MCO-0.2 catalysts were measured by scanning electron microscope (SEM, Zeiss Gemini 300) equipped with energy dispersive spectrometer (EDS, Oxford X-MAX). The surface elements and chemical valences of pure Co_3_O_4_, MCO-0.1 and MCO-0.2 catalysts were analysed by X-ray photoelectron spectroscopy (XPS, Thermo Scientific ESCALAB 250, USA) with Al Ka radiation, and the binding energies were calibrated with the residual C 1s peak (284.8 eV). Zeta potentials of MCO-0.2 catalysts were measured by Malvern Zeta sizer Nano ZS90 through the method described in Text S2. Electron paramagnetic resonance (EPR) signals of active species were monitored on a Bruker A300 EPR spectrometer using TEMP or DMPO as capture agents. In addition, the concentration of THIA and probe compounds were determined by high-performance liquid chromatography (HPLC, Agilent 1260), and the details of the measurements were illustrated in Text S3. Cobalt dissolution amount was measured by inductively coupled plasma-mass spectrometry (ICP-MS, Optima 5300 DV, USA). The residual concentrations of PMS were analyzed through the method described in Text S4.

## 3. Results and discussion

### 3.1. Characterization of MCO catalysts

**Fig. 1:**
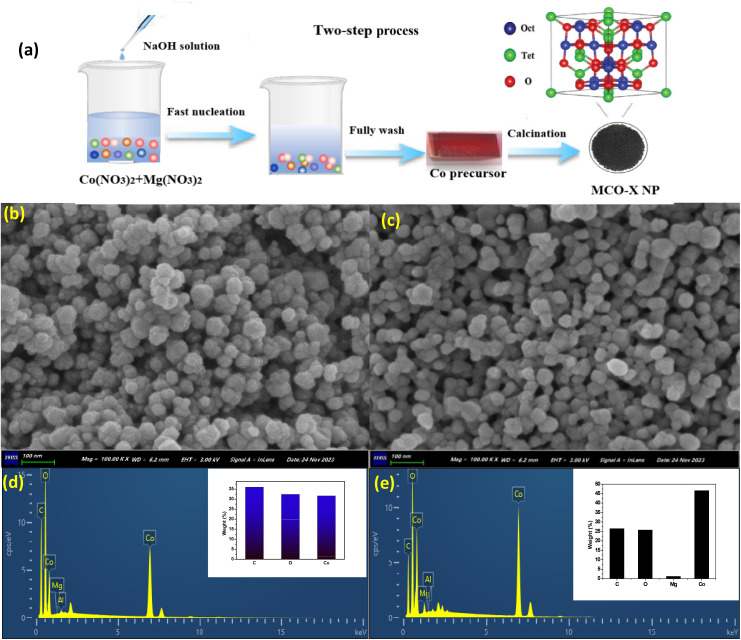
(a) Schematic illustration of the preparation of MCO-*X* catalysts, SEM images of (b) Co_3_O_4_ and (c) MCO-0.2; EDS spectra for (d) Co_3_O_4_ and (e) MCO-0.2, respectively.

MCO catalysts were prepared through a co-precipitation method followed by the calcination in air (Fig. 1a), in which the precipitate was recovered by the centrifugation at 10 000 rpm and then resuspended in DI water, repeating the sequence several times to obtain the mono-dispersed catalysts. By adjusting the amounts of Mg ions in the precursor, the Mg doping degree could be controlled and a series of MCO catalysts were obtained. SEM images of Co_3_O_4_ and MCO-0.2 catalysts were presented in Fig. 1b and c, suggesting Co_3_O_4_ exhibited the granular structure with an average diameter around 40 nm. Notably, MCO-0.2 exhibited a similar size and shape to that of Co_3_O_4_, which indicated that Mg doping did not destroy the sample morphology. Moreover, EDS analysis (Fig. 1d and e) demonstrated the presence of Co, O and Mg elements in MCO-0.2, and Mg/Co atomic ratio was about 1 : 23, suggesting partial Co could be substituted by Mg atoms. These results suggested that MCO catalysts with similar morphology and different Mg contents were successfully synthesised, which facilitate us to judge the important role of Mg doping in PMS activation.

**Fig. 2:**
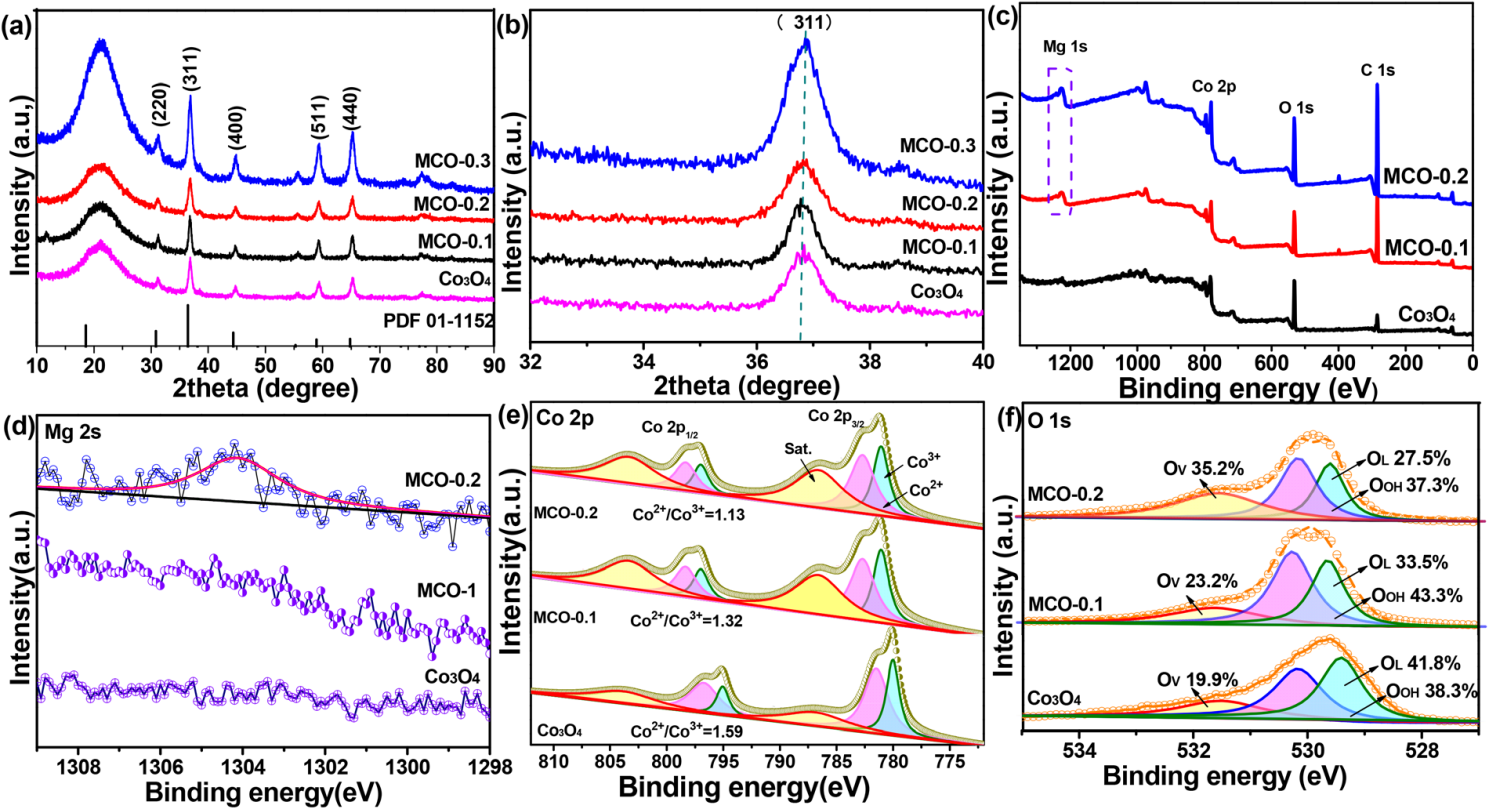
(a) XRD patterns and (b) the diffraction peak at 36–37.5° expanded in the abscissa; (c) XPS survey spectra, high-resolution XPS spectra of (d) Mg 2s, (e) Co 2p, (f) O 1s of Co_3_O_4_, MCO-0.1 and MCO-0.2 catalysts, respectively.

XRD measurements were employed to verify the crystal characteristics of Co_3_O_4_ and MCO-*X* catalysts. As can be seen in Fig. 2a, XRD pattern of Co_3_O_4_ exhibited the diffraction peaks at 2*θ* values of 18.9°, 31.3°, 36.9°, 38.4°, 44.8°, 55.7°, 59.2°, and 65.2° were attributed to (111), (220), (311), (222), (400), (422), (511) and (440) lattice planes of the spinel-type Co_3_O_4_ (space group *Fd*3̄*m*, JCPDS card no. 42-1467).^9,13^ For MCO catalysts, only peaks of Co_3_O_4_ can be observed, indicating that Mg atoms should incorporate into the Co_3_O_4_ crystal lattice and the introduction of Mg did not change the Co_3_O_4_ host lattice, due to similar ionic radius of Mg^2+^ to that of Co^2+^ (0.72 Å of Mg^2+^*vs.* 0.74 Å of Co^2+^).^17,28^ After all, Mg^2+^ has a little smaller ionic radius to that of Co^2+^, Mg doping might lead to a slight change of peak positions and intensities for these catalysts.^28,29^ Specially, a slight shift of the peak representing the (311) plane to lower angle can be observed by expanding the abscissa (Fig. 2b) of the XRD patterns for the MCO-*X* catalysts in comparison with that of the pure Co_3_O_4_. Since the (311) lattice plane of MgCo_2_O_4_ is lower to that of Co_3_O_4_ by about 2*θ* = 0.047°,^13^ the shift should be caused by the substitution of tetrahedral Co^2+^ with Mg^2+^. As Mg/Co atomic ratio increased, a further shift to the lower angle appeared on the series of MCO-*X* catalysts, indicating more tetrahedral Co^2+^ could be substituted by Mg^2+^. This structure change might lead to the improved chemical properties and the higher activity.^13^

XPS analysis was performed to probe surface elements and chemical states of pure Co_3_O_4_, MCO-0.1 and MCO-0.2 catalysts. The survey spectra (Fig. 2c) clearly confirmed the presence of Co, O and Mg elements in MCO-0.1 and MCO-0.2 catalysts, and the corresponding Mg 2p spectra (Fig. 2d) exhibited a characteristic peaks of Mg 2p, testifying the Mg atoms might be incorporated into the crystal structure of Co_3_O_4_, which agreed well with XRD measurements. In the Co 2p spectra (Fig. 2e), two main peaks at 779.4 and 794.5 eV should be attributed to Co 2p_3/2_ and Co 2p_1/2_, respectively, along with their corresponding satellite peaks (denoted as “sat.”) at 788.1 and 803.1 eV. The Co 2p_3/2_ spectra were fitted into Co^2+^ and Co^3+^ constituents at 779.7 and 781 eV, while Co 2p_1/2_ were also separated into the same components at 725.8 and 727.45 eV respectively.^36,37^ The ratio of their fitted peak area revealed that Co^2+^/Co^3+^ atomic ratio gradually decreased with the increment of Mg content, verifying Mg doping could be a potential strategy for manipulating surface metal state of Co_3_O_4_ toward improved activities. Meanwhile, the O 1s spectra (Fig. 2f) displayed three peaks, which ascribed to lattice oxygen (O_L_, ∼529.5 eV), surface hydroxyl groups (O_OH_, ∼530.3 eV), and oxygen vacancies (O_v_, ∼531.6 eV).^38^ Notably, the peak area ratio of deficient oxygen gradually increased with increased Mg content, confirming Mg doping might create new oxygen vacancies.^39^ The finding revealed that due to the lower Co^2+^/Co^3+^ atomic ratio induced by Mg doping, the neighbouring oxygen atom might be more easily oxidized by octahedral Co^3+^ and squeezed out of the crystalline structure, thus forming MCO-0.2 with enriched oxygen vacancies at the surface.^40^

### 3.2. Catalytic activity of MCO catalysts

**Fig. 3:**
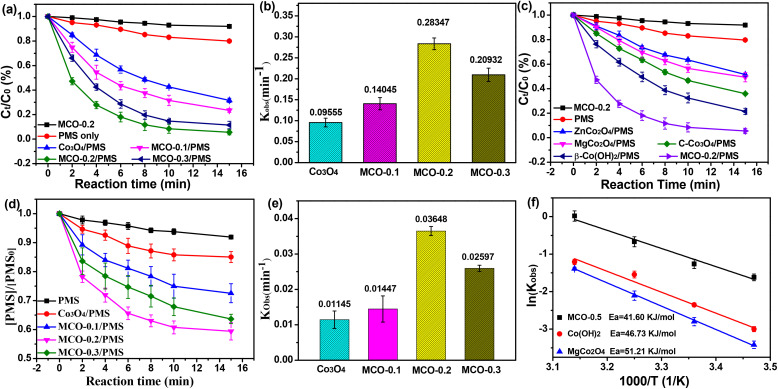
(a) THIA removal and (d) PMS depletion over Co_3_O_4_ and MCO-*X* catalysts, (b) and (e) the corresponding first-order rate constants; (c) THIA removal curves over MCO-0.2 and other similar catalysts, (f) the corresponding Arrhenius plots. Experiment conditions: pH = 7, [THIA] = 20 mg L^−1^, [catalyst] = 100 mg L^−1^, [PMS] = 0.4 mM.

The catalytic activities of MCO catalysts were evaluated in thiacloprid (THIA) degradation *via* PMS activation. Fig. 3a showed negligible THIA removal was observed when each catalyst or PMS was used separately. Optimization experiments for the catalysts were also determined. Fig. 3b showed a volcano-like relationship between the catalytic performance and Mg doping amount. MCO-0.2 exhibited the best catalytic performance for THIA degradation and the rate constants was 0.28347 min^−1^, which was about 2.97, 2.02 and 1.35 times higher than that of Co_3_O_4_ (0.09555 min^−1^), MCO-0.1 (0.14045 min^−1^) and MCO-0.3 (0.20932 min^−1^), respectively. On the other hand, PMS decomposition (Fig. 3d and e) on the surface of different catalyst was in line with THIA degradation, which could be attributed to the fact that the catalyst accelerated the PMS activation to produce more ROS, thus resulting in significant THIA degradation. The depletion rates constants of PMS in MCO-0.3, MCO-0.2, MCO-0.1 and Co_3_O_4_ systems were 0.02597, 0.03648, 0.01145 and 0.01447 min^−1^, respectively. The above results suggested the important function of Mg doping in tuning the Co^2+^/Co^3+^ ratio for enhanced THIA degradation. MCO-0.2 might have the rational Co^2+^/Co^3+^ ratio, which facilitate the Co^2+^/Co^3+^ recycle, thus promoting PMS activation toward a significantly improved THIA degradation. However, the excessive substitution of Co^2+^ with Mg in MgCO-0.3 might deteriorating the Co^2+^/Co^3+^ recycle, ultimately resulting in the decrease of degradation efficiency.^17^

To further verify the excellent activation performance of MCO-0.2 catalyst toward PMS, Similar catalysts such as β-Co(OH)_2_ and ZnCo_2_O_4_ were used to activate PMS for THIA degradation. As reported,^35,41^ the majority of cobalt was Co^2+^ ions at the tetrahedral sites of β-Co(OH)_2_, while most of cobalt was Co^3+^ ions at the octahedral sites of ZnCo_2_O_4_. Under the same conditions, the activation performance of these two catalysts (Fig. 3c and S1c) was considerably lower than that of MCO-0.2, with THIA removals of 78.7.2% and 48.2%, respectively, which suggested that the rational Co^2+^/Co^3+^ ratio in MCO-0.2 catalysts could favour Co^2+^/Co^3+^ recycle and boost the PMS activation toward a rapid THIA degradation. Furthermore, in comparison with commercial Co_3_O_4_ (denoted as C-Co_3_O_4_) and MgCo_2_O_4_ under the identical conditions (Fig. 3c and S1c), it was observed that the MCO-0.2 exhibited the higher THIA removal rates, which also highly outperformed many other reported cobalt-based catalysts (Table S2), indicating that MCO-0.2 had the extremely superior ability of PMS activation and great application potential. More interestingly, the activation energies (*E*_a_) derived from a series of kinetic experiments also exhibited the similar trend (Fig. 3f and S2). The activation energy (*E*_a_) of the MCO-0.2/PMS system was calculated to 41.6 kJ mol^−1^ through Arrhenius formula, which was considerably lower than that of Co(OH)_2_/PMS (46.7 kJ mol^−1^) and ZnCo_2_O_4_/PMS (51.2 kJ mol^−1^) systems, indicating that the rational Co^2+^/Co^3+^ ratio in MCO-0.2 catalyst could notably promote THIA degradation by lowering *E*_a_. Thus, we selected MCO-0.2, which displayed the superior PMS activation capability, as the catalyst for further experiments.

### 3.3. Versatile applicability and reusability of MCO-0.2

**Fig. 4:**
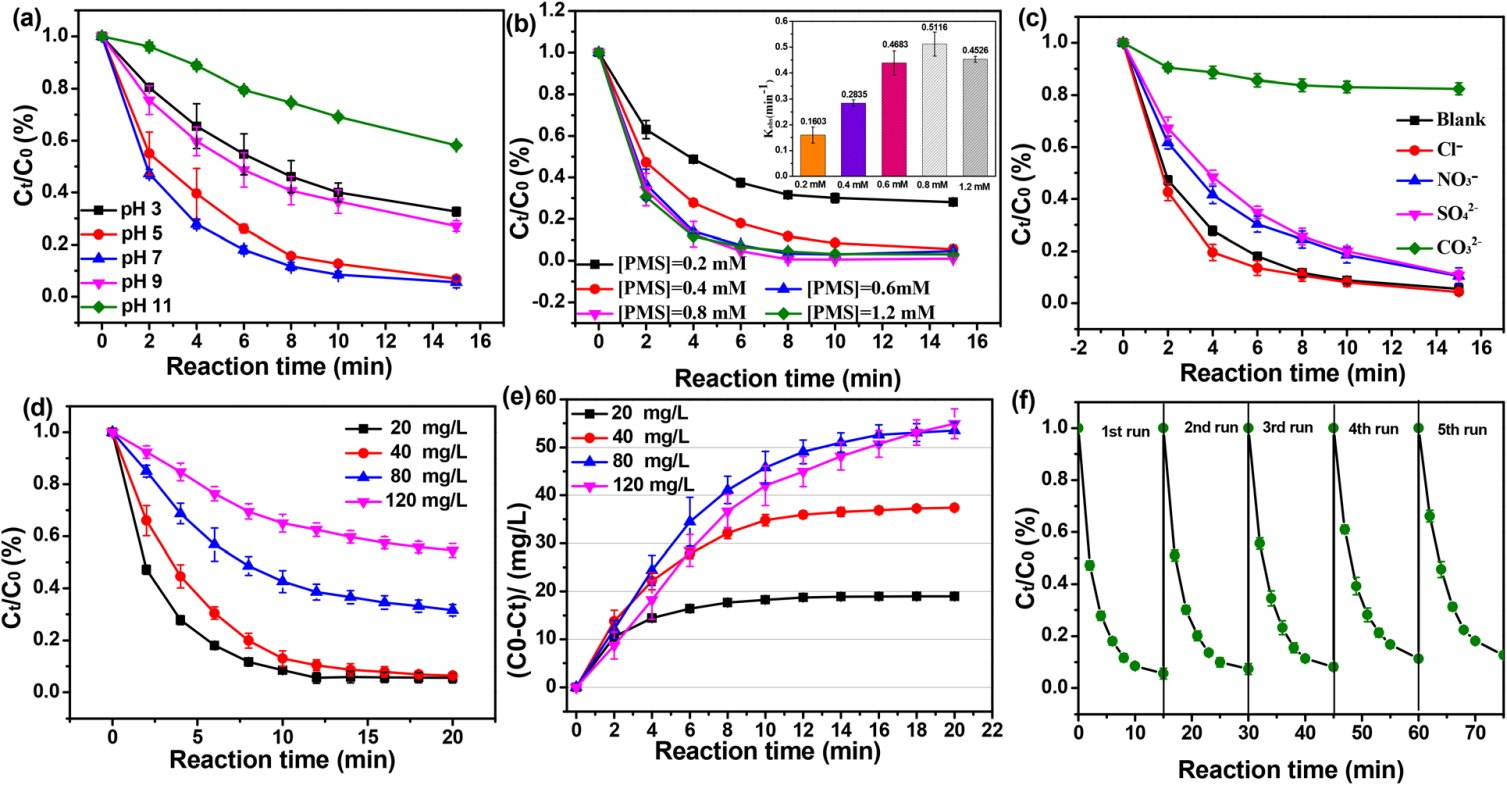
Effects of (a) initial pH, (b) PMS concentration, (c) coexisting ions and (d) and (e) THIA concentration on THIA degradation in the MCO-0.2/PMS system; (f) THIA degradation in the recycle tests of MCO-0.2 catalysts. Reaction conditions: [THIA] = 20 mg L^−1^, [PMS] = 0.4 mM, [catalyst] = 100 mg L^−1^, *T* = 298 K, pH = 7, [Cl^−^]_0_ = [NO_3_^−^]_0_ = [HCO_3_^−^]_0_ = [SO_4_^2−^]_0_ = 100 mM.

To investigate the universal applicability of MCO-0.2, THIA degradation in the MCO-0.2/PMS system were examined under different reaction conditions (*e.g.*, initial pH, PMS dosage, THIA concentration and co-existing anions). Fig. 4a showed the activity of MCO-0.2 was susceptible to the changes of pH value. The neutral pH condition achieved a higher THIA removal efficiency, while either acidic conditions or alkaline condition achieved a relatively low THIA degradation. Specifically, THIA removal efficiency was increased from 67.9% to 94.4% in 15 min by increasing the initial pH from 3.0 to 7.0. While THIA removal efficiency significantly declined from 94.4% to 41.9% with the further increase of pH from 7.0 to 11.0. As reported, p*K*_a_1__ and p*K*_a_2__ of PMS were about 0 and 9.4, respectively, which indicated that the main form of PMS was HSO_5_^−^ under the acidic conditions and neutral conditions, while the main form was SO_5_^2−^ under alkaline conditions.^42,43^ Zero potential of MCO-0.2 was 6.91 (Fig. S3), demonstrating that the catalyst surface was mainly positively charged under negatively charged under alkaline conditions. As a result, pH has significant impact on the adsorption and existence form of PMS, thus notably affecting PMS activation. In the acidic solution, HSO_5_^−^ and RS could be severely scavenged by excessive H^+^, therefore hampering PMS activation and THIA degradation. Under alkaline conditions, the main form of PMS was SO_5_^2−^ under alkaline conditions, which could be more difficultly activated to produce RS than that one (HSO_5_^−^) under acidic condition and neutral condition. Furthermore, the electrostatic repulsion between the negatively charged catalyst and SO_5_^2−^ also greatly restrained the adsorption of PMS on the catalyst surface, thus future blocked PMS activation.

As for PMS dosage (Fig. 4b), the degradation efficiency of THIA was sharply enhanced as PMS dosage increased from 0.2 to 0.8 mM. However, the enhancement was negligible with PMS dosage further increasing. The optimal dosage of PMS was 0.8 mM with respect to practical cost. Apparently, more PMS could generate more ROS in the reaction system and accelerate THIA degradation. However, excessive PMS might quench the active radicals in the reaction system and deteriorate THIA degradation. Similar results were also reported by Li and Gong's groups.^44,45^Fig. 4d showed THIA removal efficiency decreased from 94.4% to 40.3% as the concentration of THIA increased from 20 to 120 mg L^−1^, and the rate constants (Fig. S4b) decreased from 0.2835 to 0.04475 min^−1^, which could be attributed to insufficient RS produced from constant PMS to pollutants. Notable, although the reaction rates were different with the higher initial concentration of THIA (80 mg L^−1^*vs.* 120 mg L^−1^), the final removal amount (*C*_0_–*C*) was almost the same (Fig. 4e), suggesting that the removal of one THIA might consumed approximately two PMS molecules for these concentrations.

Some anions (including Cl^−^, HCO_3_^2−^, NO_3_^−^, and SO_4_^2−^) might be naturally present in actual water environment and affect THIA degradation.^46,47^ Therefore, the impact of these co-existing anions on THIA degradation was investigated in the MCO-0.2/PMS process. As shown in Fig. 4c, NO_3_^−^ and SO_4_^2−^ possessed slight negative impact on THIA degradation with degradation efficiencies from 94.4% to 89.6% and 89.1% in 15 min, respectively, manifesting NO_3_^−^ and SO_4_^2−^ might react with reactive species (SO_4_˙^−^ and/or ˙OH) to generate the radicals with weaker redox, thus inhibit THIA degradation.^46^ While THIA degradation efficiencies increased from 94.4% to 95.8% after Cl^−^ was spiked the system, implying the reaction of Cl^−^ and reactive species might convert into powerful radicals (such as Cl_2_˙^−^ and ClO˙),^48^ and future boost THIA degradation. Additionally, HCO_3_^−^ caused a significant restraint (https://fanyi.so.com/) on THIA removal with degradation efficiencies decline to 17.8% in 15 min, which might be attributed to the fact that HCO_3_^−^ could easily scavenge reactive species (SO_4_˙^−^ and/or ˙OH) SO_4_˙^−^ and/or ˙OH to generate weak oxidant (CO_3_˙^−^), which attacks THIA more slowly.^49^ THIA degradation in various water conditions (such as deionized water, tap water and lake water) was also investigated. As shown in Fig. S9, similar THIA removal rates were witnessed in deionized water (DI water) and tap water. In lake water, THIA removal rate exhibited an apparent inhibition but still exceeded 85%, suggesting excellent practical application prospects.

Finally, the reusability of MCO-0.2 catalysts was evaluated through successive THIA degradation tests. Fig. 4f showed that the degradation rates of THIA declined slightly and were still above 85% after five cycles. THIA degradation rate for the five recycles was 94.4%, 92.8%, 91.2, 88.8% and 87.3% in 15 min, and the rate constant (*k*) was estimated to be 0.2835, 0.2668, 0.2427, 0.2121 and 0.19297 min^−1^ (Fig. S5b), demonstrating superior reusability of MCO-0.2. Simultaneously, the leaching of cobalt ion from MCO-0.2 catalysts was tracked during the successive processes. The dissolved cobalt ions for the five recycles was 0.082, 0.065, 0.062, 0.057 and 0.056 mg L^−1^ (Fig. S5c), which were all far below the permissible discharge quantity for surface water in China.^3^ In addition, THIA degradation was carried out in dissolved Co ions (Co^2+^ 0.08 mg L^−1^)/PMS system (Fig. S6) and THIA degradation efficiency was only 7.17%, indicating the leached cobalt ions contributed little to THIA removal. The above results prove MCO-0.2 catalysts have the excellent and stable catalytic activity just with a slight decrease after five cycles. The negligible decrease of the catalyst activity might be ascribed to the accumulation of products or intermediates on the catalyst during the THIA degradation process and cobalt leaching from the catalyst in the four recycling processes.

### 3.4 Mechanistic insights into PMS activation by MCO-0.2

#### 3.4.1 Identification of reactive species

While it is generally agreed that various RS (including SO_4_˙^−^, ˙OH, ^1^O_2_) were involved in the PMS-AOPs process, but their relative importance for pollutant abatement still remained significant controversies.^10^ Originally, sulfate radical (SO_4_˙^−^) and its secondary radical (hydroxy radical, ˙HO) were proposed as the primary reactive intermediates for pollutant abatement during the persulfate-based process.^50–52^ Recently, a series of increasing finding demonstrated that ^1^O_2_ was the dominant reactive intermediates and played an even more important role than SO_4_˙^−^ and ˙OH for pollutant abatement, especially during PMS-AOPs activated with carbon-based materials and carbon-metal composites.^53–55^ However, latest studies^56,57^ showed high-valent cobalt-oxo (Co(iv)=O) species were successfully detected in the PMS-AOPs by the fact that Co(iv)=O could easily oxidize DMSO to the corresponding sulfone product PMSO_2_, and contributed greatly to pollutant abatement_._ Thus, it is crucial to delve deeper into the components of the MCO-0.2/PMS system, which dominated the reaction process.

**Fig. 5:**
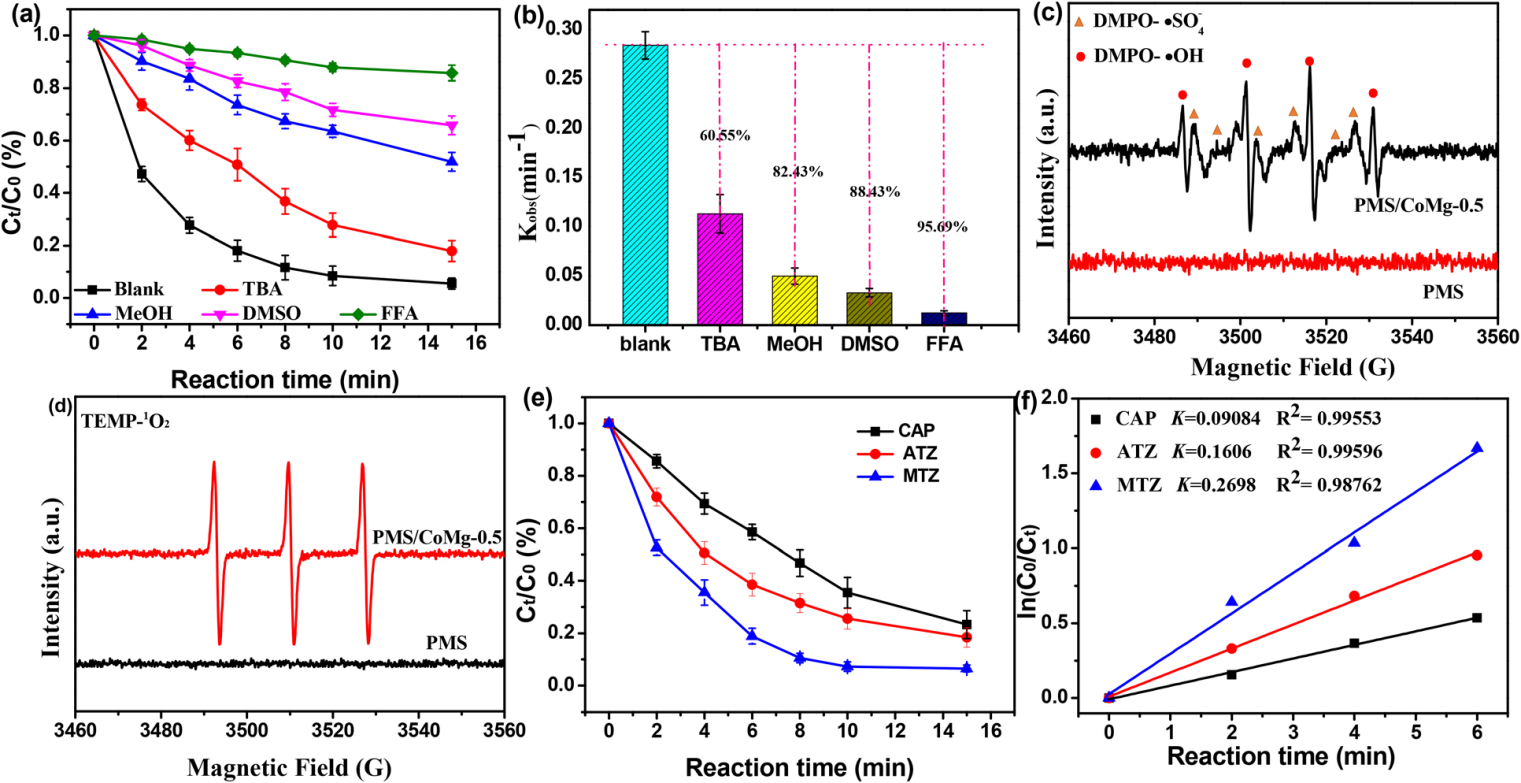
(a) Removal efficiencies of THIA on MCO-0.2 catalysts in the presence of 200 mM quenchers, and (b) the corresponding first-order rate constants; EPR spectra of (c) DMPO and (d) TEMP as the trapping agent in the MCO-0.2/PMS system; (e) abatement of probe compounds on MCO-0.2 catalysts, and (f) the corresponding kinetic curves. Reaction conditions: [catalysts] = 100 mg L^−1^, [PMS] = 0.4 mM, [THIA] = 20 mg L^−1^, [CAP] = [ATZ] = [MTZ] = 0.08 mM, pH = 7, *T* = 298 K.

First, quenching experiments were firstly conducted to prove the presence of ROS during the MCO-0.2/PMS process. MeOH was used as the scavengers of sulfate radical (SO_4_˙^−^) and hydroxy radical (˙HO). While TBA, FFA and DMSO were applied to scavenge ˙OH, ^1^O_2_ and Co(iv) =O, respectively. As reported, MeOH can synchronously scavenge ˙OH or SO_4_˙^−^ (*k*(MeOH, ˙OH) = 9.7 × 10^8^ M^−1^ s^−1^; *k*(MeOH, SO_4_˙^−^) = 3.2 × 10^6^ M^−1^ s^−1^), but TBA can merely efficiently capture ˙OH (*k*(TBA, ˙OH) = 6 × 10^8^ M^−1^ s^−1^; and *k*(TBA, SO_4_˙^−^) = 8.4 × 10^5^ M^−1^ s^−1^).^58^Fig. 5a and b showed TBA and MeOH could apparently inhibited THIA degradation and the degradation rate decreased from 94.3% to 81.21% and 48.21% in 15 min, respectively, confirming the generation of SO_4_˙^−^ and ˙HO in THIA degradation. While FFA could almost completely inhibited THIA degradation, and the corresponding rate constant (*k*) decreased to 0.0122, accounting for a 95.69% decrease, which confirmed the generation of ^1^O_2_ radicals. Further, the introduction of DMSO displayed a remarkably promoted inhibition for THIA degradation, and reduced the reaction rate by 63.76%. However, the formation of PMSO_2_ could not catch in the process. Since the reaction of PMSO with ˙OH or SO_4_˙^−^ could also result in a vigorous inhibitory for THIA abatement without the formation of PMSO_2_,^59^ it is reasonable to speculate that Co(iv)=O was almost absent in the reaction system.

Furthermore, EPR tests were performed to verify the formation of the above-mentioned ROS in MCO-0.2/PMS system. Fig. 5c and d showed no distinct characteristic peaks were detected when only PMS was introduced in the THIA solution, indicating that the generation of ROS from PMS self-decomposition was negligible. However, when PMS and MCO-0.2 were added simultaneously in the system, characteristic peaks of different ROS could be clearly observed. To be specific, Fig. 5c showed the four well-defined peaks with intensity ratio of 1 : 2 : 2 : 1 were ascribed to the characteristic signals of DMPO-˙OH adducts, while the rest peaks were accordant with typical character signals of DMPO-SO_4_˙^−^ adducts, signifying the formation of ˙OH and SO_4_˙^−^ during the MCO-0.2/PMS process. In Fig. 5d, the three-line signals with an intensity ratio of 1 : 1 : 1 were observed, ascribing to the characteristic signals of ^1^O_2_, confirmed the presence of ^1^O_2_ in the THIA degradation process. Therefore, EPR measurement further identified the involvement of ˙OH, SO_4_˙^−^ and ^1^O_2_ in THIA degradation, agreeing well with the results of quenching tests.

#### 3.4.2. Relative contributions of various reactive species

The above results proved that SO_4_˙^−^, HO˙ and ^1^O_2_ were the reactive oxidants in the MCO-0.2/PMS system. Therefore, THIA degradation kinetic in the system could be simulated as follows:^60^3
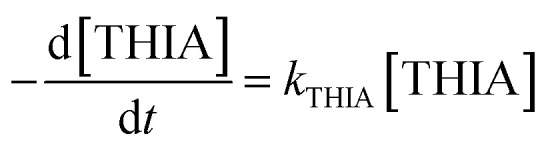


Integrating eqn (3) and yields eqn (4).4
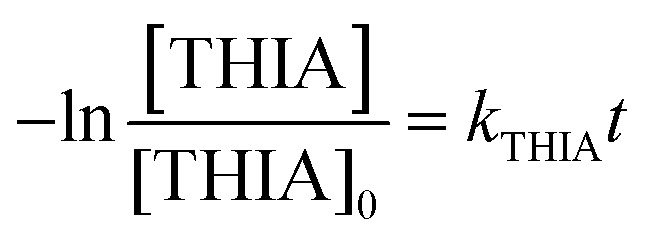
5
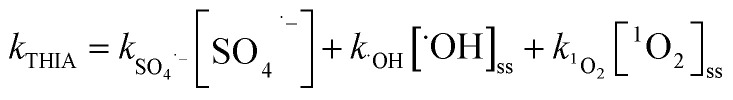
where [THIA]_0_ and [THIA] were the concentration of THIA at time 0 and *t*, respectively. 
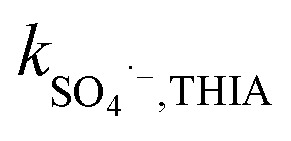
, *k*˙_OH,THIA_ and *k*_^1^O_2_,THIA_ were the second-order rate constant for the reaction of THIA with SO_4_˙^−^, HO˙ and ^1^O_2_, respectively (Table S2). *k*_THIA_ was the pseudo-first-order rate constant of THIA degradation in the MCO-0.2/PMS system. [SO_4_˙^−^]_ss_, [˙OH]_ss_ and [^1^O_2ss_] represented the steady-state concentrations of SO_4_˙^−^, HO˙ and ^1^O_2_, respectively, and could be measured by several probe experiments.

In the probe experiments, probe compounds (including CAP, ATZ and MTZ) were added in the MCO-0.2/PMS system under identical conditions, and the abatement kinetic of these probes could be expressed as follows:6
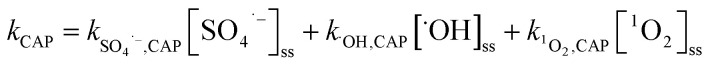
7
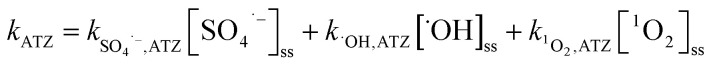
8
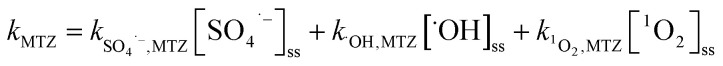
where *k*_CAP_, *k*_ATZ_ and *k*_MTZ_ were the pseudo-first-order rate constants of the degradation of CAP, ATZ and MTZ probes in the MCO-0.2/PMS system, respectively, which were obtained from the kinetic plots in Fig. 5e and f. Moreover, 
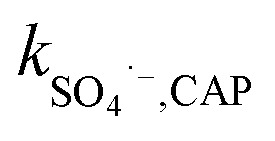
, *k*˙_OH,CAP_, 
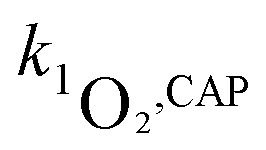
, 
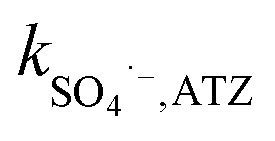
, *k*˙_OH,ATZ_, *k*_^1^O_2_,ATZ_, 
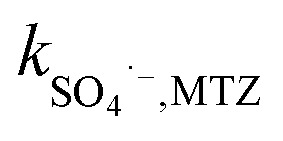
, *k*˙_OH,MTZ_ and *k*_^1^O_2_,ATZ_ represented the second-order reaction rate constants of SO_4_˙^−^, HO˙ and ^1^O_2_ with CAP, ATZ and MTZ, respectively (Table S1). By using the eqn (6)–(8), the steady-state concentrations of ^1^O_2_, ˙OH and SO_4_˙^−^ in the MCO-0.2/PMS system were thus readily calculated to be 2.321 × 10^−10^, 1.079 × 10^−11^ and 4.408 × 10^−11^ M, respectively.

Further, the relative contributions of SO_4_˙^−^, HO˙, ^1^O_2_ and other potential reactive species were defined as the ratios of THIA degradation rate induced by one specific reactive intermediate to the total degradation rate of THIA (*k*_THIA_),^16^ and could be expressed as follows:9
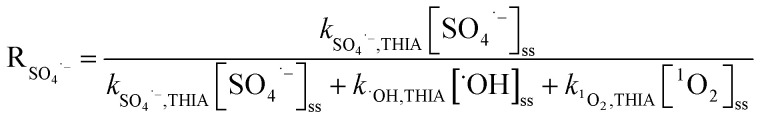
10
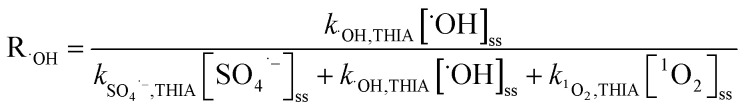
11
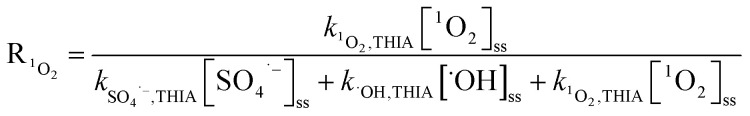
12
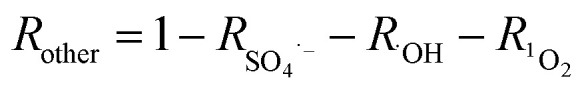


Using the newly measured kinetic data and the eqn (6)–(8), the relative contributions of SO_4_˙^−^, HO˙, ^1^O_2_ and other potential reactive species for 
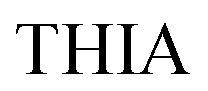
 degradation in the MCO-0.2/PMS system were quantified to be 17.2%, 74.1%, and 3.5%, respectively. The measured results implied the contribution of ˙OH, SO_4_˙^−^ and ^1^O_2_ to THIA abatement relied on both their reactivity and exposures. As reported,^61^
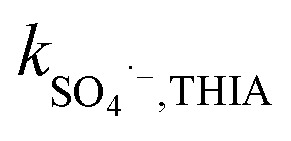
 and *k*˙_OH,THIA_ were several orders of magnitude larger than *k*_^1^O_2_,THIA_. Therefore, although the exposures of ˙OH/˙SO_4_^−^ are about two or more times lower than that of ^1^O_2_, they dominated THIA abatement in the system. Further, since ˙OH exposures are 2.9 times higher than the exposures of SO_4_˙^−^, it contributed predominantly to THIA abatement in the MCO-0.2/PMS system given the similar value of 
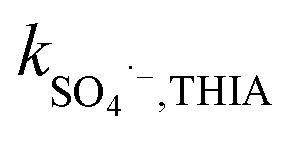
 and *k*˙_OH,THIA_.

Combined with the above analysis, a possible activation mechanism of PMS on MCO-0.2 catalysts was proposed (Fig. S8). Initially, PMS was adsorbed onto the catalyst surface and activated by the active Co^2+^, accompanying with the generation of Co^3+^ and SO_4_˙^−^; meanwhile Co^3+^ ions withdraw electrons from PMS to form Co^2+^ and SO_5_˙^−^, driving a Co^2+^/Co^3+^ cycle. Subsequently, partial SO_4_˙^−^ further react with H_2_O or OH^−^ of the solution to produce HO˙. SO_4_˙^−^ and HO˙ would also react with PMS to produce SO_5_˙^−^, which could be decomposed into ^1^O_2._ Consequently, upon a rapid Co^2+^/Co^3+^ cycle, these generated RS could efficiently degraded THIA molecules over MCO-0.2 catalysts, where HO˙ might contributed mainly to THIA degradation given the exposures of these RS and their reactivity with THIA.

## 4. Conclusions

Mg-doped Co_3_O_4_ with the regulated ratios of Co^2+^/Co^3+^ were prepared by incorporating Mg dopants into the lattice of Co_3_O_4_ and used as PMS activator for THIA degradation. Structural characterizations and experimental investigations confirmed that Mg doping did not change the Co_3_O_4_ host lattice and particle morphology, but could manipulate surface metal state of Co_3_O_4_ for an improved PMS activation. The optimized sample (MCO-0.2) with the suitable Co^2+^/Co^3+^ atomic ratios (1.13) exhibited efficient THIA degradation, and the rates constants (0.28347 min^−1^) highly outperformed that of pure Co_3_O_4_, β-Co(OH)_2_ and ZnCo_2_O_4_. A systematic analysis of the influences of reaction parameters indicated that the maximum degradation might be achieved under the conditions: catalyst dose 100 mg L^−1^, PMS concentration 0.8 mM, pH 7 and THIA concentration 20 mg L^−1^. Quenching experiment and ERP tests confirmed that ^1^O_2_, SO_4_˙^−^ and ˙OH were all involved in MCO-0.2/PMS System. According to a kinetic model and a series of probe compound-based experiments, the steady-state concentrations of SO_4_˙^−^, HO˙ and ^1^O_2_ were determined to be 1.079 × 10^−11^, 4.408 × 10^−11^ and 2.321 × 10^−10^ M in MCO-0.2/PMS system, and their corresponding contributions to THIA degradation were also quantified to 17.2%, 74.1%, and 3.5%. This work may inform a new approach for constructing efficient Fenton-like catalysis.

## Conflicts of interest

There are no conflicts to declare.

## Supplementary Material

RA-015-D5RA04080A-s001

## Data Availability

All data included in this study are available upon request by contact with the corresponding author. Additional details on analytical methods of PMS and various organic pollutants, SI degradation results and the parameters for various organic pollutants, and other associated SI tables and figures. See DOI: https://doi.org/10.1039/d5ra04080a.
