# Supplementary material for: Efficient peroxymonosulfate activation by magnesium-doped Co3O4 for thiacloprid degradation: regulation of Co2+/Co3+ ratios and degradation mechanism
Source: RSC Adv. 2025 Aug 28;15(37):30817–28. doi: 10.1039/d5ra04080a (PMC12395025; doi:10.1039/d5ra04080a)
Supplement: RA-015-D5RA04080A-s001 [file RA-015-D5RA04080A-s001.pdf]

Supplementary Information (SI) for RSC Advances.
This journal is © The Royal Society of Chemistry 2025

Supporting Information

Efficient peroxymonosulfate activation by magnesium-doped Co_3O_4 for thiacloprid degradation: Regulation of Co^{2+}/ Co^{3+} ratios and degradation mechanism

Hui Fui,*[a,b] Xinran Ma,[b] Yiping Huang,[b] Shiyao Xi,[b] Zhandong Ren[a,b] and Yuchan Zhu[a,b]

[a] Hubei Province Key Laboratory of Agricultural Waste Resource Utilization, [b] School of Chemistry and Environmental Engineering, Wuhan Polytechnic University, Wuhan430023, China

*Corresponding author: HUi Fui: Feihui509@163.com

Text S1. Reuse test.

To collect and separate the products in the MCO-0.2/PMS process, the reaction volume was increased by 10 times to 1 L of reaction solution containing 100 mg catalyst, 20 mg thiacloprid, 0.4 mmol PMS. After 15 min, the solution was quenched with $Na_2S_2O_3$ solution and centrifuged to separate the solid and liquid. The solid products were collected and washed with DI and ethanol for three times before the next run.

To track cobalt leaching amount among the reuse tests, sample was withdrawn and quenched with $Na_2S_2O_3$ solution after each run. Then the quenched samples were centrifuged to separate the solid and liquid. The supernatants were collected and analyzed by Inductively Coupled Plasma-Mass Spectrometry.

Text S2. Determine the pH point of zero charge (pHzpc).

Typically, 50 mL of 0.1 M KCl solution at six different pH (3, 5, 7, 9, 11) and 0.01 g of MCO-0.2 catalysts were applied to the determination of pHzpc. Then place the prepared solutions on the shaker for 24 h and measured the final pH after 24 h. $\Delta pH = pH_{final} - pH_{initial}$ was calculated and its curve plotted as the X = initial pH and Y = ΔpH. The intersection of the curve with the X-axis is equal to pHzpc.

Text S3. Quantification of various pollutants.

The concentration of THIA, ATZ, CAP and $PMSO_2$ were determined by high performance liquid chromatography (HPLC, Agilent 1260) equipped with a 179 4.6×250 mm column. The mobile phases (v/v) were formic acid (1‰)/acetonitrile (40/60, v/v) for THIA, water/methanol (50/50, v/v) for ATZ, water/methanol (90/10, v/v) for CAP, water/acetonitrile (30/70, v/v) for $PMSO_2$. THIA, ATZ, CAP and $PMSO_2$ were measured at 242, 357, 278, 265 and 265 nm, respectively. MTZ concentration was analyzed with a UV–vis spectrophotometer (UV-3600, Shimadzu, Japan) at 319 nm.

Text S4. Quantification of PMS.

PMS concentration was analyzed using KI spectrophotometry.[1] Briefly, 2 mL of suspension was withdrawn at given time intervals and filtered with a 0.22-μm polytetrafluoroethylene (PTFE) syringe filter immediately. Then, 1.0 mL of filtered sample was added to 4 mL of KI solution (0.5 M, containing 0.05 M $NaHCO_3$). After reacting for 30 min, the mixture was analyzed by a UV-visible absorption spectrometer (UV-3600, Shimadzu, Japan) at 352 nm.

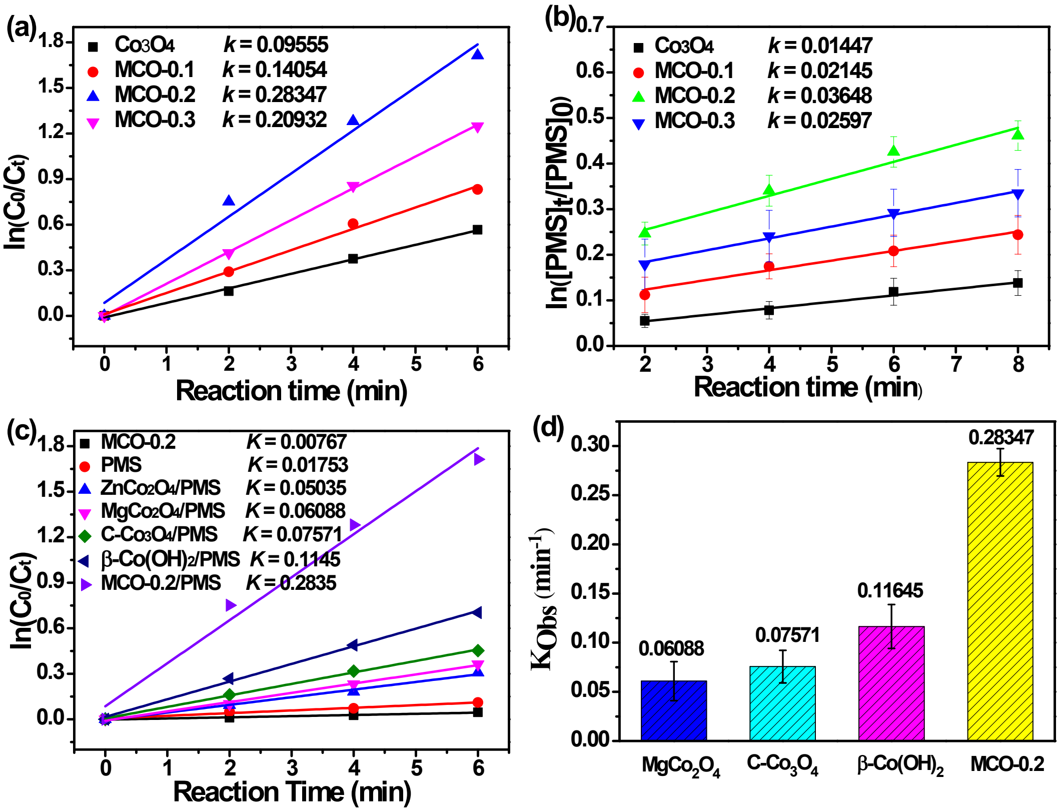

Figure S1. Kinetic curves of (a) THIA removal and (b) PMS depletion over Co_3O_4 and MCO-x catalysts; (c) Kinetic curves of THIA removal over MCO-0.2 and other similar catalysts, (d) the corresponding first-order rate constants. Reaction conditions: [THIA] = 20 mg/L, [catalyst] = 100 g/L, [PMS] = 0.4 mM, T = 298 K, pH = 7.

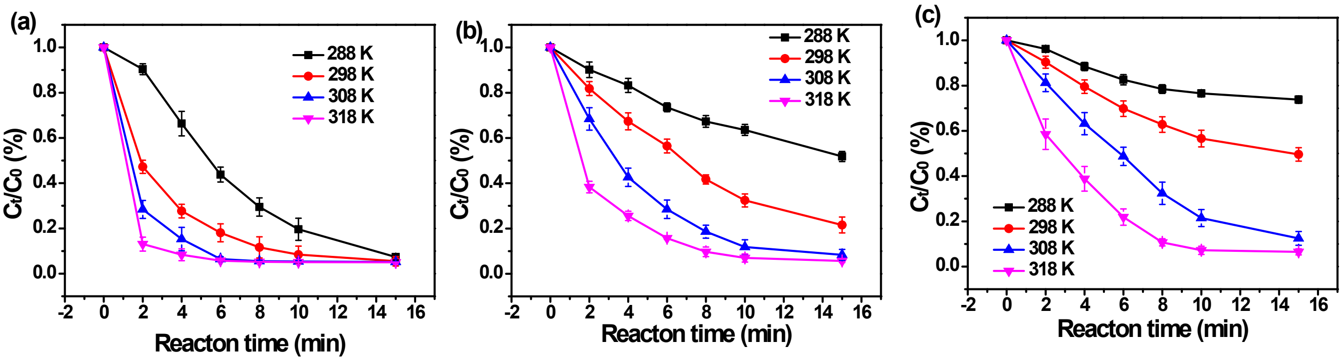

Figure S2. Kinetic curves of THIA removal obtained from different temperatures in (a) MCO-0.2/PMS, (b) β-$Co(OH)_2$/PMS and (c) $ZnCo_2O_4$/PMS system. Reaction conditions: [THIA] = 20 mg/L, [catalyst] = 100 g/L, [PMS] = 0.4 mM, T = 298 K, pH = 7.

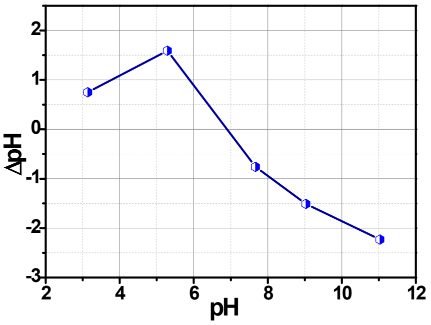

Figure S3. Zeta potential of MCO-0.2 catalysts at different pH.

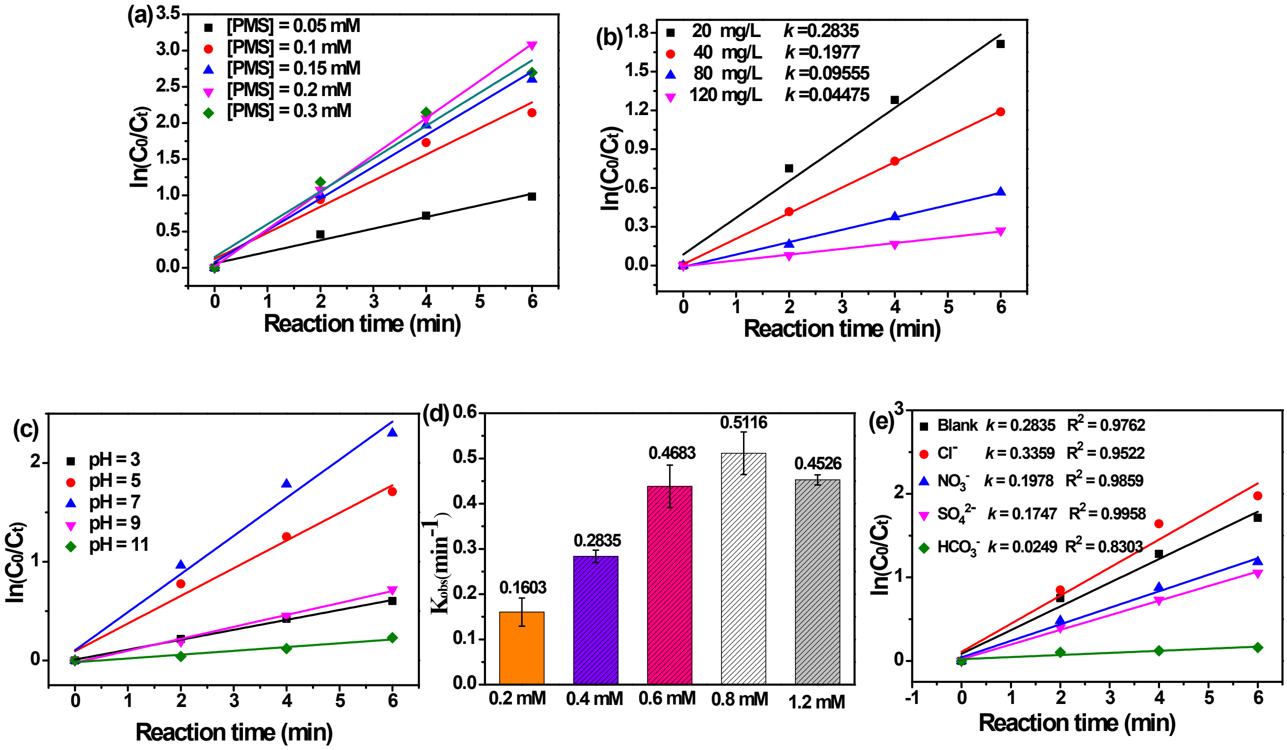

Figure S4. Kinetic curves of THIA removal at different reaction conditions: (a) PMS dosage, (b) THIA concentration, (c, d) initial pH, (e) co-existing anions. Reaction conditions: [THIA] = 20 mg L^{-1}, [PMS] = 0.4 mM, [catalyst] = 100 mg L^{-1}, T = 298 K and pH =7.

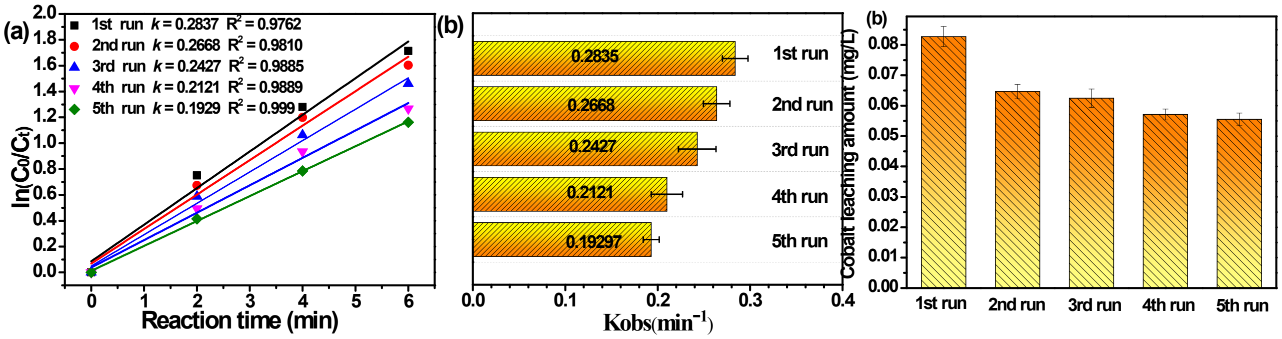

Figure S5. (a) Kinetic curves of THIA removal, (b) the corresponding rate constant and (c) Co leaching in multiple runs with MCO-0.2catalysts. Reaction conditions: [THIA] = 20 mg/L, [catalyst] = 100 g/L, [PMS] = 0.4 mM, T = 298 K, pH = 7.

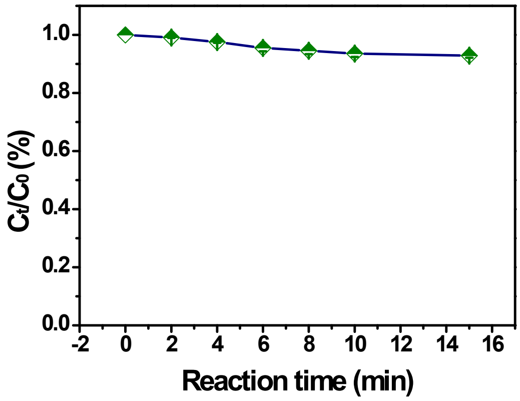

Figure S6. THIA removal during the leaching Co^{2+}/PMS process. Reaction conditions: [THIA] = 20 mg/L, [Co^{2+}] = 0.08 mg/L, [PMS] = 0.4 mM, T = 298 K, pH = 7.

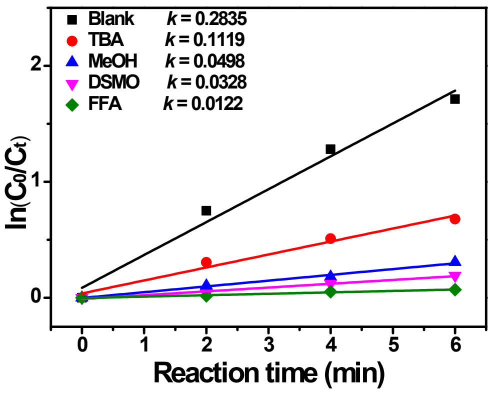

Figure S7. Kinetic curves of THIA removal in the presence of 200 mM quencher. Experimental conditions: [THIA] = 20 mg/L, [catalyst] = 100 mg/L, [PMS] = 0.4 mM, T= 298 K, pH = 7.

Table S1. The second-order rate constants for reactions of the probe contaminants and THIA with •OH, $SO_4^{•-}$, $^1O_2$ and Co(IV), respectively.

Compound	k_{PMS}	$k_{SO_4^{•-}}$	$k_{•OH}$	$k_{^1O_2}$	$k_{Co(IV)}$	Ref.
TBA	No reaction	4.0×10^5	6.0×10^8	3.04×10^3	1.0×10^2	2
MeOH	No reaction	1.1×10^7	9.7×10^8	3.89×10^3	6.1×10^3	2
FFA	0.011	1.3×10^{10}	1.5×10^{10}	1.20×10^8	No reaction	2,
DMSO	No reaction	2.7×10^9	7.0×10^9	No reaction	2.4×10^6	3
ATZ	0.04	2.6×10^9	3.0×10^9	4.0×10^4		4,5
MTZ	No reaction	2.74×10^9	3.54×10^9	3.40×10^8		6, 7
CAP	0.08	9.1×10^8	1.80×10^9	7.21×10^6		2,4
THIA	0.14	4.5×10^9	4.8×10^9	3.9×10^7		2, 8

Table S2. Comparison with reported works of other catalyst systems.

Catalyst	Reaction conditions	Removal efficiency	K_{obs}(mol^{-1})	Ref
**Iron-Doped Manganese Oxide**	[catalyst] = 0.04 g/L, [PMS] = 0.5 mM, [BPA] = 1.15 mg/L	95% in 20 min	0.0138	9
**$Fe_xCo_{3-x}O_4$**	[BPA] =20 mg/L [PMS] = 0.2g/L, [catalyst] = 0.1 g/L	95% in 60 min	0.049	10
**$CoMn_2O_4$**	[SA] = 10 mg/L [PMS] = 0.1 g/L, [catalyst] = 0.05 g/L	100% in 20 min	0.155	11
**alginate@$ZnCo_2O_4$**	[PMS] = 1 g/L, [catalyst] = 1 g/ L [RhB] = 25 mg/L	98.28% in 40 min	0.1459	12
**$Co_{2.98}Al_{0.02}O_4$**	[MNZ] = 10 mg/L, [catalyst] =0.5 g/L, [PMS] = 1.5 mM	100% in 40 min	0.127	13
**MCO-0.2**	[THIA] = 20 mg/L, [catalyst] =100 mg/L, [PMS] = 0.4 mM	94.4% in 15 min	0.2835	**This work**

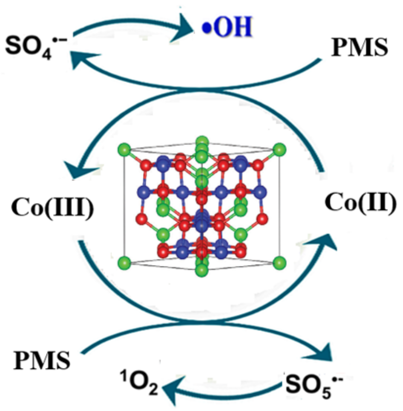

Figure S8. Possible activation mechanism of PMS in MCO-0.2/PMS system.

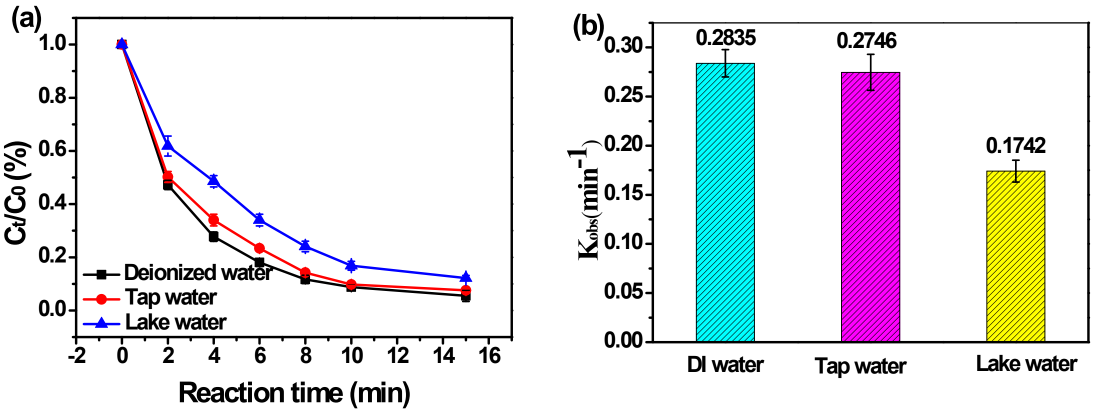

Figure S9. THIA removal in various water conditions. Reaction conditions: [THIA] = 20 mg/L, [Co^{2+}] = 0.08 mg/L, [PMS] = 0.4 mM, T = 298 K, pH = 7.

References

1. C. J. Liang, C. F. Huang, N. Mohanty and R. M. Kurakalva, A rapid spectrophotometric determination of persulfate anion in ISCO, Chemosphere, 2008, 73,1540-1543.

2. L. Gao, Y. Guo, J. Zhan, G. Yu and Y. Wang, Assessment of the validity of the quenching method for evaluating the role of reactive species in pollutant abatement during the persulfate-based process, Water Res., 2022, 221, 118730.

3. Y. Zong, X. H. Guan, J. Xu, Y. Feng, Y. F. Mao, L. Q. Xu, H. Q. Chu and D. L. Wu, Unraveling the Overlooked Involvement of High-Valent Cobalt-Oxo Species Generated from the Cobalt(II)-Activated Peroxymonosulfate Process, Environ. Sci. Technol., **2020,** *54* (24), 16231-16239.

4. L. Gao, Y. Guo, J. Huang, B. Wang, S. Deng, G. Yu and Y. Wang, Simulating micropollutant abatement during cobalt mediated peroxymonosulfate process by probe-based kinetic models, Chem. Eng. J., 2022, 441, 135970.

5. L. Gao, Y. Guo, J. Zhan, G. Yu and Y. Wang, Assessment of the validity of the quenching method for evaluating the role of reactive species in pollutant abatement during the persulfate-based process, Water Res., 2022, 221, 118730.

6. L. Lian, B. Yao, S. Hou, J. Fang, S. Yan and W. Song, Kinetic study of hydroxyl and sulfate radical-mediated oxidation of pharmaceuticals in wastewater effluents, Environ. Sci. Technol., 2017, 51(5), 2954–2962.

7. Y. Guo, J. Long, J. Huang, G. Yu and Y. Wang, Can the commonly used quenching method really evaluate the role of reactive oxygen species in pollutant abatement during catalytic ozonation? Water Res., 2022, 215, 118275.

8. G. R'ozsa, L.Szab'o, K. Schrantz, E. Tak'acs, L. Wojn'arovits, Mechanistic study on thiacloprid transformation: Free radical reactions. J. Photoch, Photobio. A: Chem., 2017, 343, 17–25.

9. K. Z. Huang and H. C. Zhang, Direct Electron-Transfer-Based Peroxymonosulfate Activation by Iron-Doped Manganese Oxide (δ-MnO_2) and the Development of Galvanic Oxidation Processes (GOPs), Environ. Sci. Technol., 2019, 53, 12610-12620.

10. X. Li, Z. Wang, B. Zhang, A. I. Rykov, M. A. Ahmed and J. Wang, $Fe_xCo_{3-x}O_4$ nanocages derived from nanoscale metal–organic frameworks for removal of bisphenol A by activation of peroxymonosulfate, Appl. Catal. B-Environ., 2016, 181, 788–799.

11. C. X. Li, C. B. Chen, J. Y. Lu, S. Cui, J. Li, H. Q. Liu, W. W. Li and F. Zhang, Metal organic framework-derived $CoMn_2O_4$ catalyst for heterogeneous activation of peroxymonosulfate and sulfanilamide degradation, Chemical Engineering Journal, 2018, 337, 101-109.

12. B. E. Channab, M. E. Ouardi, S. E. Marrane, O. A. Layachi, A. E. Idrissi, S. Farsad, D. Mazkad, A. l. BaQais, M. Lasri and H. A. Ahsaine, Alginate@$ZnCO_2O_4$ for efficient peroxymonosulfate activation towards effective rhodamine B degradation: optimization using response surface methodology, RSC Adv., 2023, 13, 20150–20163.

13. H. T. Li, Q. Gao, G. S. Wang, B. Han, K. S. Xia, J. P. Wu, C. G. Zhou and J. Dong, Postsynthetic incorporation of catalytically inert Al into Co_3O_4 for peroxymonosulfate activation and insight into the boosted catalytic performance, Chemical Engineering Journal, 2021, 426, 131292.
